# p53-R273H promotes cancer cell migration via upregulation of neuraminidase-1

**DOI:** 10.7150/jca.44718

**Published:** 2020-10-04

**Authors:** Tao Lv, Hong Lv, Junjie Fei, Yajun Xie, Daqing Lian, Jiang Hu, Lizhou Tang, Xiaodong Shi, Jianling Wang, Shibo Zhang, Fengtian Li, Xianjie Jiang, Yong Yi

**Affiliations:** 1 Center for Yunnan Plateau Biological Resources Protection Utilization, College of Biological Resource and Food Engineering, Qujing Normal University, Qujing, Yunnan, China 655011.; 2 Hematology Department, The First People's Hospital of Qujing, Qujing, Yunnan, China 655000.; 3 Center of Growth, Metabolism and Aging, and Key Laboratory of Bio-Resource and Eco-Environment, Ministry of Education, College of Life Sciences, Sichuan University, Chengdu, China 610064.; 4 The Ministry of Education Key Laboratory of Laboratory Medical Diagnostics, the College of Laboratory Medicine, Chongqing Medical University, Chongqing, China 400016.; 5 College of Chemistry and Environmental Science, Qujing Normal University, Qujing, Yunnan, China 655011.; 6 The Key Laboratory of Carcinogenesis and Cancer Invasion of Ministry of Education, Cancer Research Institute, Central South University, Changsha 410078, China.

**Keywords:** p53-R273H, NEU1, ITGB4, AKT, cell migration

## Abstract

Accumulating evidence indicates that hotspot p53 mutants have gain-of-function in promoting cell migration and tumor metastasis. However, the molecular mechanisms are not completely understood. Here, we show that a hotspot mutation, p53-R273H, promotes non-small cell lung cancer (NSCLC) cell migration and upregulates the mRNA and protein expression of neuraminidase-1 (NEU1), a sialidase involved in cell proliferation, cell migration and tumorigenesis. Silencing of NEU1 leads to upregulation of integrin β4 which significantly inhibits NSCLC cell migration induced by p53-R273H. Mechanistically, p53-R273H promotes NEU1 transcription via activation of AKT signaling. Importantly, NEU1 expression is upregulated in human NSCLC samples harboring mutant p53 and is associated with poor clinical outcome. Overall, this study highlights an important role of NEU1 in p53-R273H-induced NSCLC cell migration and provides a potential target for NSCLC diagnosis and treatment.

## Introduction

Lung cancer is the leading cause of cancer-related mortality and histologically is classified into small-cell lung cancer (SCLC) and non-small cell lung cancer (NSCLC). NSCLC accounts for approximately 80%-85% of all lung cancers 1, 2. Mounting evidence indicates that proto-oncogenes, such as *RAS*,* EGFR* or *PIK3CA*, are often activated in NSCLC 3-5. Moreover, the *p53* tumor suppressor gene, the most studied gene in the human genome 6, is frequently mutated in NSCLC 7, 8. Accumulating clinical evidence indicates that mutant p53 is associated with NSCLC poor clinical outcome and cancer metastasis 9, 10.

The p53 protein contains five distinct domains, which include an N-terminal transactivation domain (TAD), a proline-rich domain (PRD), a DNA binding domain (DBD), an oligomerization domain (OD), and a C-terminal regulatory domain (CTD) 11. Accumulating evidence indicates that p53 regulates a series of biological processes, including DNA damage repair, glucose metabolism, senescence, apoptosis and cell cycle arrest 12. Several mutations clustered within the central DBD, referred to as “hotspot” mutations, are frequently found in cancers, refereed as “hotspot” mutations, and include R273, R175, G245, R248, R249 and R282 13. It has been documented that mutant p53 loses its original function and acquires gain of function (GOF) 14. Mutant p53 can promote cancer cell survival, anti-apoptosis, metastasis, angiogenesis, and resistance to chemotherapy 15-19. At the molecular level, mutant p53 can directly bind to AMPK, the catalytic subunit of AMP-activated protein kinase (AMPK), to promote cancer cell proliferation and tumor growth 20. Moreover, mutant p53 can promote integrin recycling or inhibit tight junction marker ZO-1 expression to facilitate cancer cell invasion and metastasis 18, 21. We have previously shown that hotspot p53 mutant p53-R273H promotes the expression of Nrp2, resulting in cancer cell mobility and metastasis 17.

Neuraminidase-1 (NEU1) is a neuraminidase family protein that catalyzes the removal of sialic acid residues from the glycan chains of glycoproteins, oligosaccharides and sialylated glycolipids and modulates molecular and cellular recognition events 22, 23. It has been reported that NEU1 plays a critical role in sialidase-mediated tumorigenesis 24. Recently, it has been documented that NEU1 has a potential role in the regulation of cell proliferation, migration, invasion and cancer metastasis 25-27.

In this study, we show that mutant p53 (p53-R273H) promoted NEU1 transcription via activation of AKT which leads to downregulation of ITGB4, consequently resulting in increased NSCLC cell mobility. Silencing of NEU1 or AKT inhibitor treatment significantly inhibited p53-R273H-induced NSCLC cell migration. Together, this study highlights the role of NEU1 in mutant p53-induced NSCLC cell migration and provides a potential target for NSCLC diagnosis and treatment.

## Materials and Methods

### Cell culture and drug treatment

Human non-small cell lung cancer (NSCLC) H1299 and HEK 293T (human embryonic kidney) cells were cultured in DMEM (Gibco, Rockville, MD, USA) supplemented with 10% fetal bovine serum (FBS; HyClone, Logan, UT, USA). Human NSCLC H1975 cells were cultured in RPMI-1640 medium (Gibco) supplemented with 10% FBS (HyClone). All cells were grown in medium supplemented with 100 units/mL penicillin (Gibco) and 100 μg/mL streptomycin (Gibco). Cells were maintained in a humidified 37 °C incubator under a 5% CO_2_ atmosphere. Cells at 75-85% confluence were treated with the indicated chemical compound. MK2206 (S1078) was purchased from Selleck Chemicals (Houston, USA).

### Plasmid transfection and lentiviral infection

Cells at 70-80% confluence were transfected using Lipofectamine 2000 transfection reagent (Invitrogen). Recombinant lentiviruses were amplified by co-transfecting HEK 293T cells with the psPAX2 and pMD2.G packaging plasmids and p53-R273H, NEU1 or ITGB4 lentiviral expression plasmid using Lipofectamine 2000. Viruses were collected at 60 hours after transfection. Cells at 50% confluence were infected with a recombinant lentivirus in the presence of 10 μg/mL polybrene, followed by 12 hours of incubation at 37°C with 5% CO_2_. Lentiviral-based shRNAs specific for green fluorescent protein (GFP) (GAAGCAGCACGACTTCTTC), p53 (#1 AAG-ACTCCAGTGGTAATC TACT; #2 CACCATCCACTACAACTACAT), NEU1 (#1 CCCGGAATCT CTCCCTGGATA; #2 GCTTCAGCAATGGTACCTCAT) and ITGB4 (AAGAACCGGATGCTGCTTATT) were constructed as described 28.

### Western blot analyses

Cells were lysed in EBC_250_ lysis buffer (250 mM NaCl, 25 mM Tris, pH 7.4, 0.5% Nonidet P-40, 50 mM NaF, and supplemented with 0.5 mM Na_3_VO_4_, 0.2 mM phenylmethylsulfonyl fluoride, 20 μg/mL aprotinin and 10 μg/mL leupeptin). Equal amounts of total protein were separated by SDS-PAGE, transferred to PVDF membrane and hybridized to an appropriate primary antibody and HRP-conjugated secondary antibody for subsequent detection by enhanced chemiluminescence. Antibodies to detect the following proteins were used in western blotting: p53 DO-1 (1:1000, Santa Cruz Biotechnology), NEU1 (1:1000, Abcam), Akt (1:1000, Abcam), pAkt (Ser473, 1:2000, Abcam), Integrin β4 (1:1000, 4707, Cell Signaling Technology), and GAPDH (1:2000, Cell Signaling Technology).

### Quantitative PCR

For quantitative PCR (QPCR) analysis, total RNA was isolated from cells using an RNA extraction kit (Qiagen, Germany) according to the manufacturer. Complementary DNA (cDNA) was generated using the first-strand cDNA kit (TAKARA, Inc). QPCR analyses were performed in a CFX96 Real-Time PCR System (Bio-Rad) using SoFast EvaGreen Supermix (Bio-Rad), according to the manufacturer's instructions. The reactions were carried out in a 96-well plate at 95 °C for 2 min, followed by 35 cycles of 95 °C for 15 s and 55 °C for 30 s. GAPDH was used as a reference. QPCR primers specific for GAPDH (F: TGGACTCCACGACGTACTCA; R: AATCCCATCACCATCTTCCA); NEU1 (F: TGTGACCTTCGACCCTGAGC; R: TCGCAGGGTCAGGTTCACTC); DDR1 (F:5'-CATGAGCCGGAACCTCTA-3'; R: 5'-CCACAGGGTCACACCAAA-3'); KRT8 (F:5'-CTGTCCATGAAGGATGACTT-3'; R: 5'-TGTCCACTCTGTCTGTG AGA-3'); GRAMD1B (F: 5'-TGGGGGAGAAGATTGAGATG-3' ; R: 5'-TGTCC A CGCTGAAGTTGAAG-3'); LTBP3 (F: 5'-CGGAACGGAGTGTGTGAGAA- 3'; R:5'-CTCGTCCACGTCCATCTCT-3'); TNFRSF12A (F: 5'-CTGGCTCC AGAACAGAAAGG-3'; R: 5'-GGGCCTAGTGTCAAGTCTGC-3'); and ITGB4 (F: 5'-CACCGCGTGCTAAGCACAT-3'; R: 5'-TGTGGTCGAGTGAGTGTTCTG-3') were used.

### Transwell assays for cell migration

Transwell assays were performed as described 29. Briefly, cell migration was measured using 6.5 mm, 8.0 μm-pore polycarbonate membrane transwell inserts (BD Biosciences, San Jose, CA, USA). Cells were suspended in serum-free media and seeded into the inner chamber (H1299, 0.5 × 10^5^ cells per chamber; H1975, 2.0 × 10^5^ cells per chamber). The outer chamber contained complete growth media. Cells were incubated for 24 hours. Nonmigrating cells were carefully removed with a cotton swab. Migrating cells were stained with 0.4% crystal violet in methanol for 20 min at room temperature and imaged under a Zeiss light microscope.

### Bioinformatics analysis

The Oncomine and TCGA databases were used to analyze NEU1 mRNA levels in clinical human lung cancers. The Kaplan-Meier plotter platform (https://kmplot.com/analysis/) was used to analyze the overall survival of lung cancer patients.

### Statistical analysis

GraphPad Prism 6.0 (GraphPad Software Inc. USA) was used for data recording, collection, processing, and calculation. ImageJ (Rawak Software, Inc. Germany) software was used to analyze cell migration. All experiments were performed at least three times in triplicate. Quantitative data were analyzed statistically using Student's *t*-test to assess significance.

## Results

### p53-R273H promotes NEU1 expression

**Figure 1:**
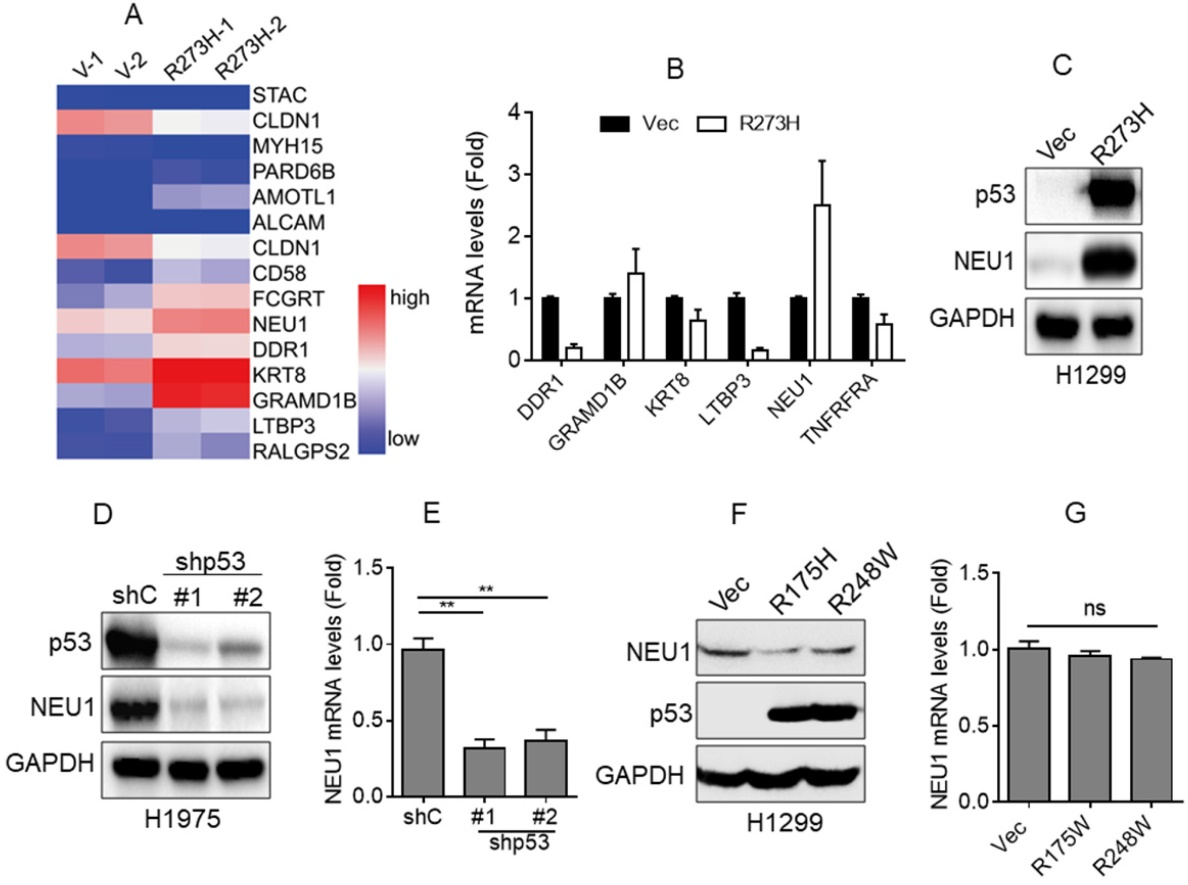
** p53-R273H promotes NEU1 expression. (A)** H1299 cells stably expressing vector control (Vec) or mutant p53-R273H (R273H) were subjected to RNA-seq analyses in duplicate. A heatmap showing changes of differentially expressed genes related to cell migration. **(B-C)** H1299 cells stably expressing Vec or R273H were subjected to Q-PCR (B) or Western blot (C) analyses.**(D-E)** H1975 cells stably expressing shGFP (shC) or shRNA specific for p53 (shp53-1, shp53-2) were subjected to Western blot (D) or Q-PCR analyses (E). **(F-G)** H1299 cells stably expressing Vec, p53-R175H or p53-R238W were subjected to Western blot (F) or Q-PCR (G) analyses. Data are presented as means ± SEM. **, *p*< 0.01.

**Figure 2:**
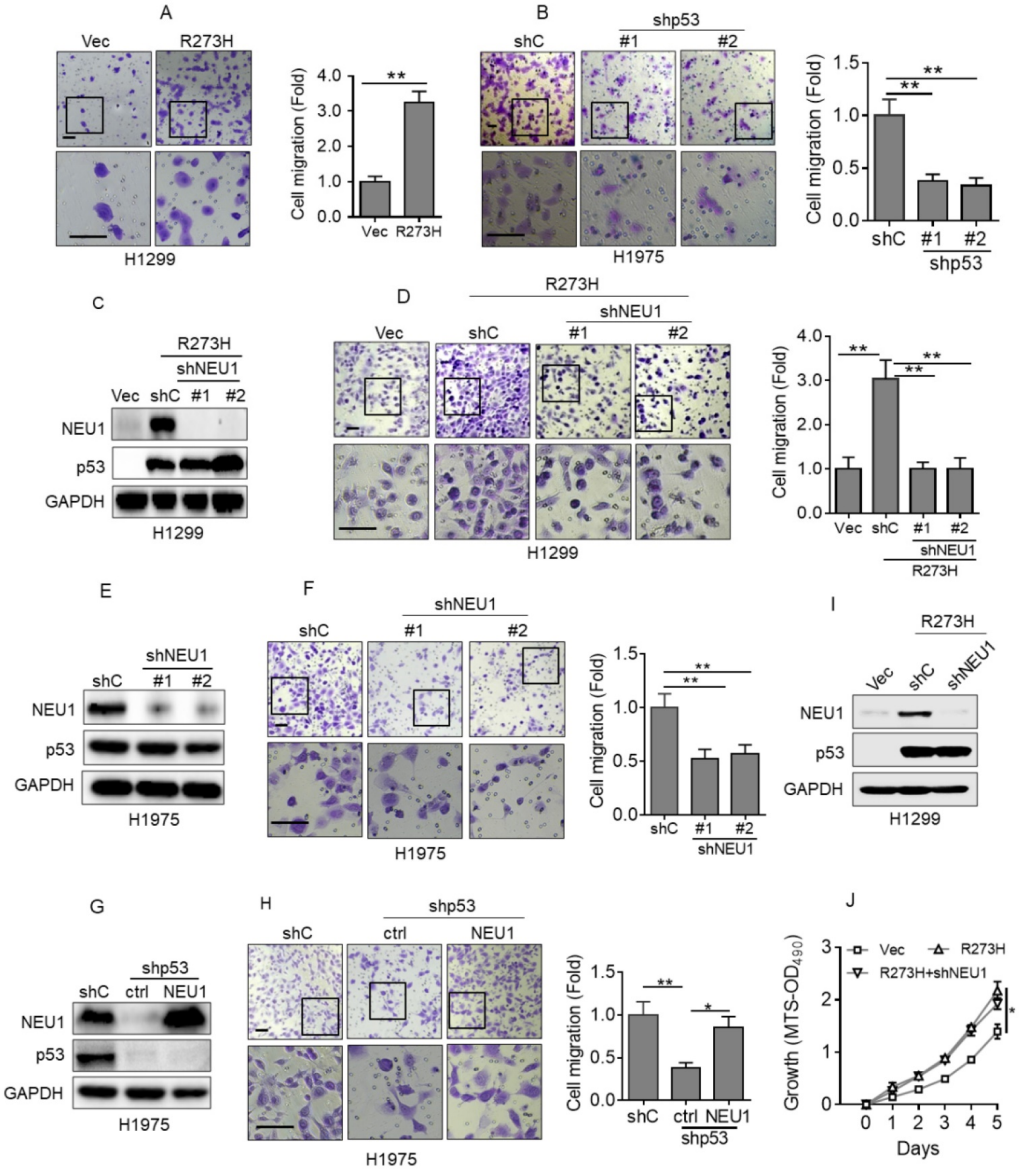
** p53-R273H promotes cell migration via upregulation of NEU1. (A)** H1299 cells stably expressing Vec or R273H (H1299-R273H) were subjected to transwell assay for cell migration.** (B)** H1975 cells stably expressing shC, shp53-1 or shp53-2 were subjected to transwell assay for cell migration.** (C-D)** H1299-R273H cells stably expressing a control shRNA (shC) or shRNA specific for NEU1 (shNEU1-1 or shNEU1-2) were subjected to Western blot analyses(C) or to transwell assay for cell migration(D). **(E-F)** H1975 cells stably expressing shC, shpNEU1-1 or shpNEU1-2 were subjected to Western blot analyses or to transwell assay for cell migration.** (G-H)** H1975-shp53 cells stably expressing NEU1 were subjected to Western blot (G) or to transwell assay for cell migration (H).** (I-J)** H1299-R273H cells stably expressing shC or shNEU1 were subjected to Western blot analyses (I) or to MTS assay for cell growth ability (J). Data are presented as means ± SEM. Scale bar =100 µm. **, *p*< 0.01; *, *p*< 0.05.

Accumulating evidence indicates that mutant p53, such as R273H or R175H, plays a critical role in NSCLC cell migration and cancer metastasis 18, 21. To investigate the underlying mechanism by which mutant p53 promotes NSCLC cell migration, we first performed RNA-Seq analyses. As shown in Figure 1A, overexpression of p53-R273H significantly upregulated the expression of a series of cell migration-related genes, including NEU1, KERT8, DDR1, LTBP3, and GRAMD1B, in NSCLC H1299 cells that lack endogenous p53. Furthermore, QPCR analyses showed that overexpression of p53-R273H notably increased NEU1 mRNA levels, consistent with the RNA-Seq data (Figure 2B). To further examine the effects of p53-R273H on NEU1 protein expression, we performed Western blot analyses in H1299 cells. As shown in Figure 1C, overexpression of p53-R273H also significantly increased NEU1 protein expression.

It has been reported that NSCLC H1975 cells harbor an endogenous p53-R273H mutation 30. Therefore, we wondered whether silencing endogenous p53-R273H could decrease NEU1 expression. To confirm this hypothesis, we stably expressed shRNA specific for p53 in H1975 cells. As shown in Figure 1D and 1E, knockdown of endogenous p53-R273H dramatically decreased NEU1 mRNA and protein levels.

In addition, we examined the effects of the other mutant p53, such as R175H and R248W, on NEU1 expression. As shown in Figure 1F and 1G, ectopic expression of p53-R175H or p53-R248W had little effects on NEU1 protein and mRNA expression in H1299 cells.

Together, these results indicate that p53-R273H can effectively promote NEU1 expression.

### NEU1 plays a pivotal role in p53-R273H-induced cell migration

To investigate the role of NEU1 in p53-R273H-induced NSCLC cell migration, we first examined the effects of p53-R273H on cell migration in our system. As shown in Figure 2A, overexpression of p53-R273H significantly increased H1299 cell migration, as evidenced by transwell analyses. Conversely, silencing endogenous p53-R273H in H1975 cells dramatically inhibited cell migration (Figure 2B). Next, to examine the role of NEU1 in p53-R273H-induced cell migration, we silenced NEU1 in H1299-R273H cells. As shown in Figure 2C and 2D, knockdown of NEU1 significantly inhibited H1299 cell migration induced by p53-R273H. Moreover, silencing endogenous NEU1 in H1975 cells also significantly inhibited cell migration (Figure 2E and 2F). Importantly, knockdown of endogenous p53-R273H in H1975 cells dramatically inhibited cell migration, which was significantly rescued by overexpression of NEU1 (Figure 2G and 2H). It has been documented that p53-R273H can promote cell proliferation and tumor growth 31. We therefore examined the effects of NEU1 on p53-R273H-induced cell proliferation. As shown in Figure 2I and 2J, consistent with previous report, p53-R273H can significantly increase H1299 cell proliferation; however, silencing of NEU1 had little effects on p53-R273H-induced H1299 cell proliferation. Together, these results demonstrate that NEU1 plays a critical role in p53-R273H-mediated upregulation of NSCLC cell migration.

### Integrin β4 is critical in p53-R273H-NEU1 axis-induced cell migration

**Figure 3:**
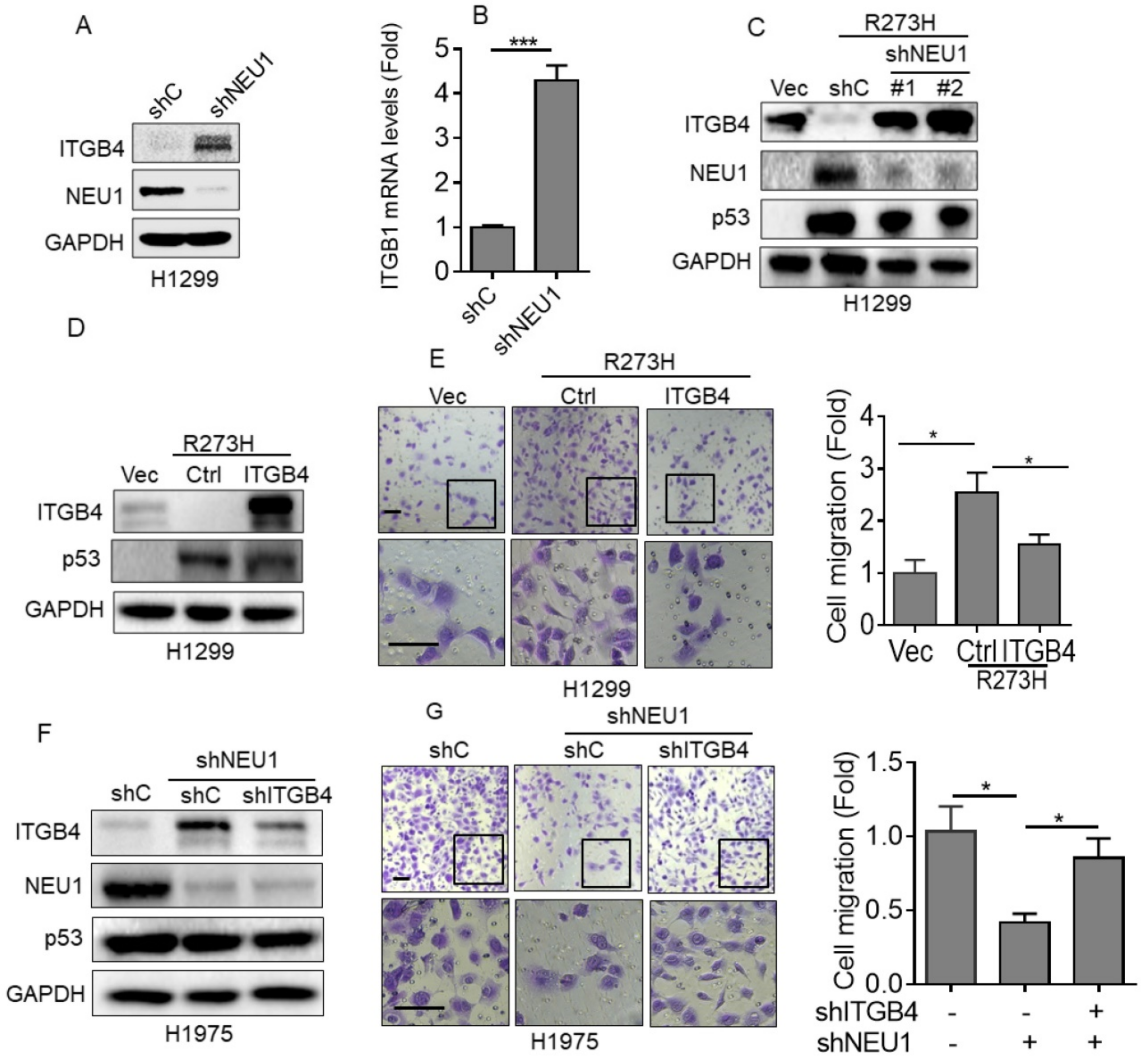
** Integrin β4 is critical in p53-R273H-NEU1 axis-induced cell migration. (A-B)** H1299 cells stably expressing shC or shNEU1 were subjected to Western blot (A) or Q-PCR (B) analyses. **(C)** H1299-R273H cells stably expressing shC, shNEU1-1 or shNEU1-2 were subjected to Western blot analyses. **(D-E)** H1299-R273H cells stably expressing integrin β4 (ITGB4) or a vector control (Ctrl) were subjected to Western blot analyses (D) or to transwell assay for cell migration (E). **(F-G)** H1975-shNEU1 cells stably expressing shC or shITGB1 were subjected to Western blot analyses (F) or to transwell assay for cell migration (G). Data are presented as means ± SEM. Scale bar =100 µm. *, *p*< 0.05.

Integrin β4 (ITGB4), an important cell-matrix adhesion molecule, plays a pivotal role in cell migration 32. It has been reported that NEU1 is critical in regulating ITGB4-mediated signaling 33. Therefore, we speculated that ITGB4 may play a role in the p53-R273H/NEU1 axis-induced NSCLC cell migration. To investigate this hypothesis, we first examined the effects of NEU1 on ITGB4 expression. As shown in Figure 3A and 3B, silencing of NEU1 significantly increased ITGB4 protein and mRNA expression. Moreover, overexpression of p53-R273H significantly increased NEU1 expression concomitant with decreased ITGB4 expression (Figure 3C). Silencing of NEU1 dramatically rescued p53-R273H-induced downregulation of ITGB4 expression (Figure 3C). Furthermore, overexpression of ITGB4 significantly inhibited p53-R273H-induced upregulation of cell migration (Figure 3D). Consistently, knockdown of NEU1 in H1975 cells significantly increased ITGB4 expression as well as inhibited cell migration (Figure 3F and 3G). Importantly, silencing of NEU1-induced downregulation of cell migration was dramatically rescued by knockdown of ITGB4 (Figure 3F and 3G). Together, these results indicate that ITGB4 plays a role in p53-R273H-NEU1 axis-induced cell migration.

### p53-R273H promotes NEU1 expression via activation of AKT signaling

**Figure 4:**
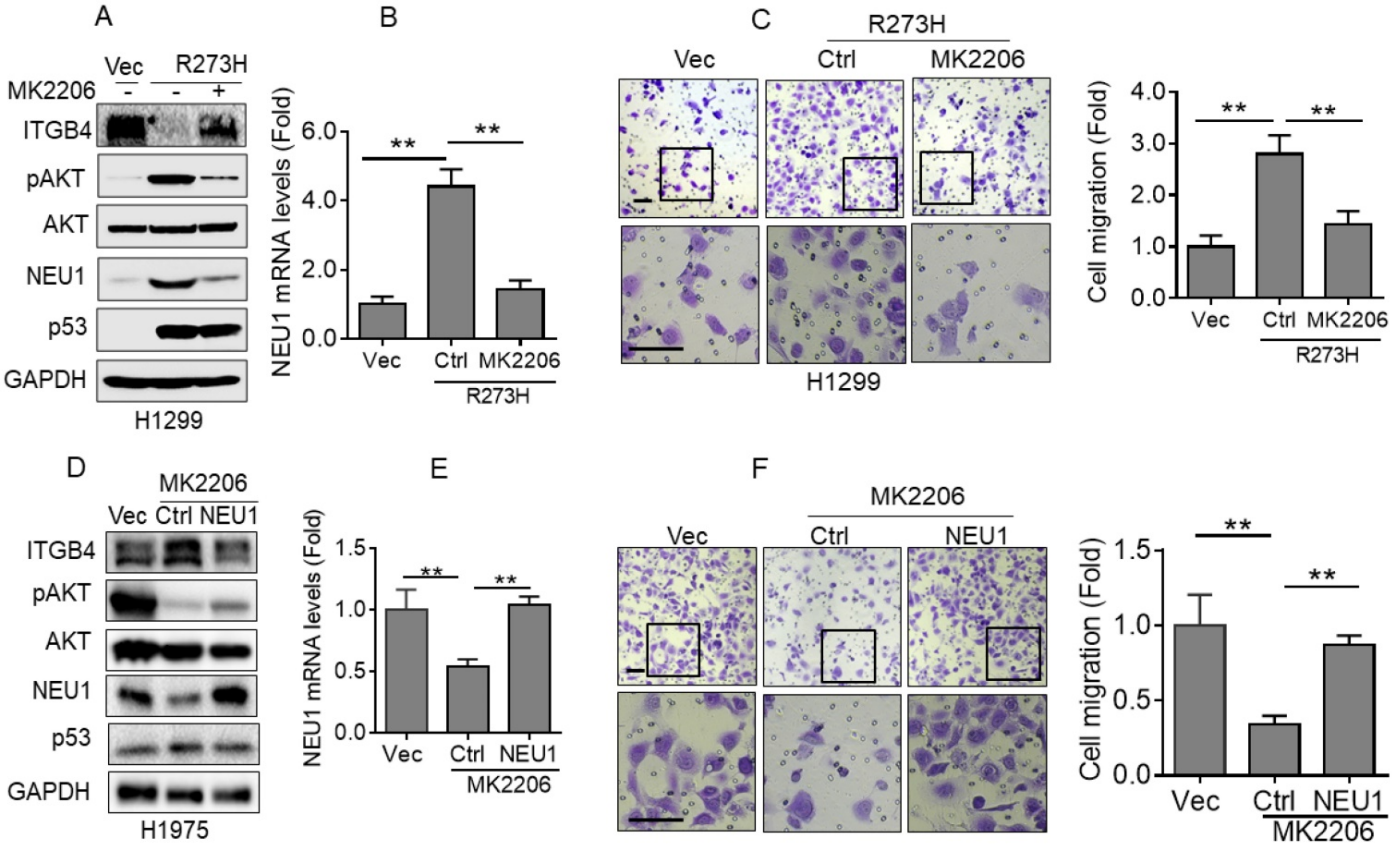
** p53-R273H promotes NEU1 transcription via activating AKT. (A-C)** H1299-R273H cells were treated or untreated with MK2206 (10 μM) for 24 h. Cells were subjected to Western blot (A) or Q-PCR (B) analyses or were subjected to transwell assay for cell migration (C). **(D-F)** H1975 cells were treated or untreated with MK2206 (10 μM) for 24 h. Cells were subjected to Western blot (D) or Q-PCR (E) analyses or were subjected to transwell assay for cell migration (F). Data are presented as means ± SEM. Scale bar =100 µm. **, *p*< 0.01.

Next, we examined the underlying mechanism by which p53-R273H promotes NEU1 expression in NSCLC cells. It has been documented that mutant p53 can activate AKT signaling by promoting epidermal growth factor receptor (EGFR) trafficking 18. Therefore, we speculated that AKT signaling may be involved in p53-R273H-mediated upregulation of NEU1 expression. To confirm this hypothesis, we used MK2206, an AKT specific inhibitor, to treat H1299-R273H cells. As shown in Figure 4A and 4B, inhibition of AKT activity significantly suppressed p53-R273H-induced NEU1 mRNA and protein expression. Moreover, p53-R273H-induced downregulation of ITGB4 protein expression also significantly was rescued by AKT inhibition. Furthermore, p53-R273H-induced H1299 cell migration was also dramatically inhibited by MK2206 treatment (Figure 4C). In addition, inhibition of AKT activity in H1975 cells also significantly inhibited NEU1 protein and mRNA levels concomitant with increased ITGB1 protein expression and decreased cell migration (Figure 4D-4F). Overexpression of NEU1 significantly rescued inhibition of AKT-induced upregulation of ITGB1 protein expression and downregulation of cell migration (Figure 4D-4F). Together, these results indicate that p53-R273H promotes NEU1 expression and cell migration through activation of AKT signaling.

### NEU1 is upregulated in human NSCLC harboring mutant p53 and is associated with poor clinical outcome

**Figure 5:**
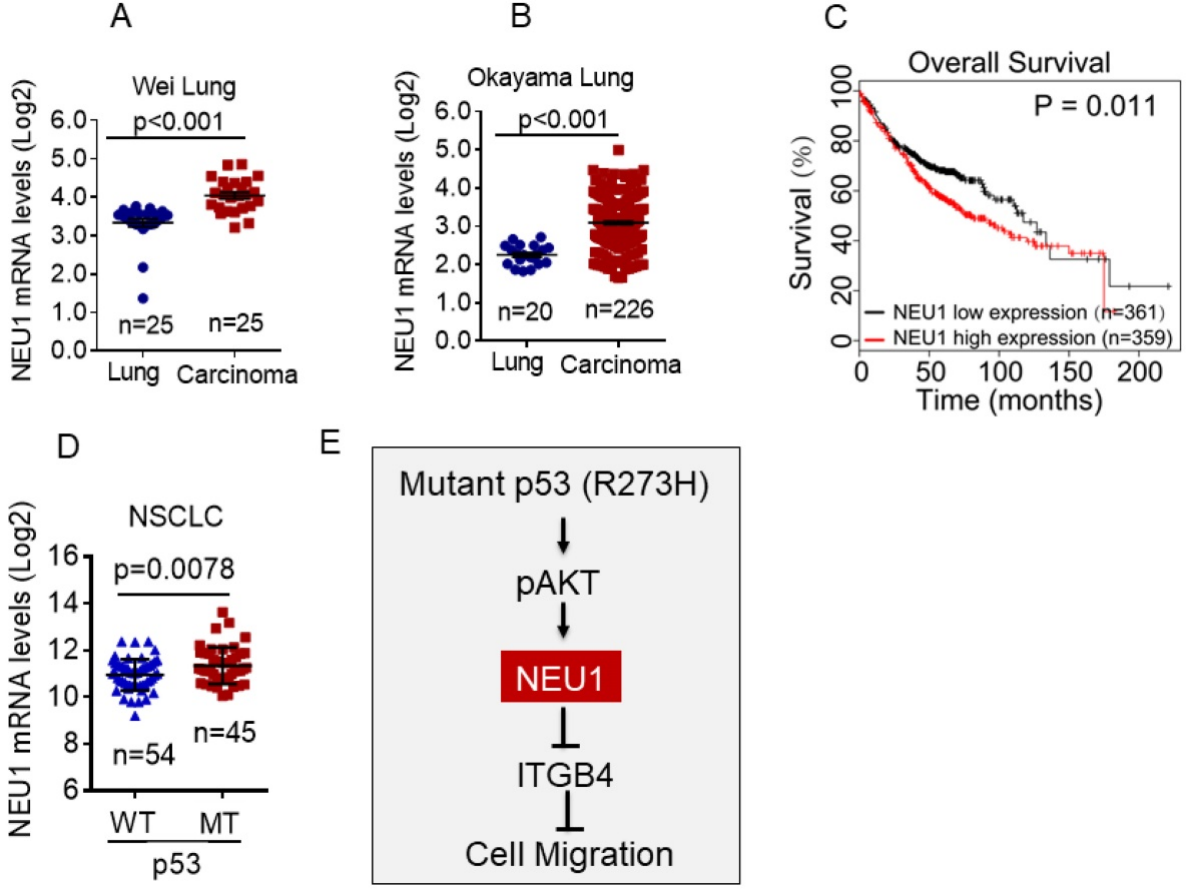
** NEU1 is upregulated in human NSCLC harboring mutant p53 and is associated with poor clinical outcome. (A-B)** The Oncomine Wei_Lung cancer dataset or Okayama lung cancer dataset was analyzed for NEU1 mRNA levels in human normal lung or lung carcinoma. **(C)** The overall survival in lung cancer patients were analyzed using Kaplan-Meier database. **(D)** The TCGA database was analyzed for NEU1 mRNA levels in human male NSCLC bearing wild type p53 alleles (p53-WT) or p53 mutant alleles (p53-MT). **(E)** A model depicts the role of NEU1 in p53-R273H-induced cell migration.

To determine the clinical relevance of NEU1 expression in human NSCLC, we first used the Oncomine database to analyze NEU1 mRNA levels in human NSCLC carcinoma tissues. As shown in Figure 5A and 5B, compared to normal lung tissues, NEU1 mRNA levels were significantly increased in NSCLC carcinoma tissues. Moreover, high NEU1 mRNA levels in NSCLC patients correlated with a short overall survival (Figure 5C). Importantly, compared to wild-type p53 NSCLC carcinoma tissues, mutant p53 NSCLC carcinoma tissues exhibited higher NEU1 mRNA expression (Figure 5D). Together, these clinical results indicate that NEU1 mRNA expression is correlated with p53 status and is associated with NSCLC patients' clinical outcome.

## Discussion

Accumulating clinical evidence indicates that *p*53 is the most commonly mutated gene in human cancer 34. It has been reported that mutant p53 is associated with poor clinical outcome in NSCLC patients 35. Mutant p53 regulates multiple biological functions, including cell proliferation, tumor growth and cancer metastasis 14, 17. It has been documented that mutant p53 can promote integrin recycling or inhibit ZO-1 expression to enhance cell invasion and cancer metastasis 18, 21. Our previous study demonstrated that p53-R273H significantly promotes cell migration and cancer metastasis via upregulation of Nrp2 expression 17. In this study, we showed that NEU1 mRNA expression is associated with NSCLC patient overall survival. NSCLC patients with high NEU1 mRNA expression exhibited low overall survival. Importantly, we found that NEU1 is a novel downstream target of p53-R273H and plays an important role in p53-R273H-induced NSCLC cell migration (Figure 5E). However, we found that the others p53 hotspot mutations, such as R175H or R248W, have little effects on NEU1 expression, suggesting that p53-R273H may specific regulate NEU1 expression.

The question remains how p53-R273H promotes NEU1 expression. It has been demonstrated that activated AKT signaling regulates a series of gene transcription pathways, including c-Myc and p63 36. Interestingly, it has been shown that mutant p53 can activate AKT signaling by enhancing EGFR trafficking 18. We showed that p53-R273H significantly promotes NEU1 transcription. Therefore, we speculate that AKT signaling may play a role in p53-R273H promoting NEU1 expression. Indeed, inhibition of AKT dramatically suppressed p53-R273H-induced NEU1 mRNA and protein expression contaminant with reduced cell migration. Our previous data indicate that AKT activation can directly phosphorylate FOXO3a, which leads to the inhibition of p63 transcription 29. However, whether FOXO3a is also involved in AKT-mediated regulation of NEU1 transcription needs to be further examined. Moreover, it is reported that p53-R273H can be used as a transcriptional co-factor binds to G/C-rich regulatory regions to active gene expression 37. There are serval G/C-rich regions on NEU1 gene promoter. Therefore, p53-R273H may regulate NEU1 transcription through both direct and indirect manners. However, whether p53-R273H direct binds to NEU1 gene promoter needs to further investigate.

NEU1 functions as a sialidase to regulate a set of genes involved in a variety of different biological processes, including cell migration and cancer metastasis 38, 39. Integrin family proteins, as cell surface adherent receptors, are fundamentally important for multiple facets of biological processes, including cell-cell junctions, metastasis and survival 18, 32, 40. Accumulating evidence indicates that integrin β4, an important integrin family protein, suppresses cancer metastasis via inhibition of EMT and promotion of cell-cell and cell-matrix adhesion 32. Interestingly, it has been reported that NEU1 plays a critical role in the regulating ITGB4-mediated signaling 33. Given these observations, ITGB4 may play a role in the p53-R273H/NEU1 axis-induced NSCLC metastasis. Indeed, our results show that ITGB4 plays a pivotal role in NEU1-induced NSCLC cell migration.

A series of clinical and basic evidence indicates that activation of EGFR plays a critical role in NSCLC initiation and development 41, 42. Targeting EGFR is a promising strategy for NSCLC treatment. Regimen EGFR tyrosine kinase inhibitor (TKI), such as erlotinib or gefitinib, exhibited a good benefit for NSCLC patients 42. However, EGFR-TKI resistance is a major challenge for NSCLC treatment 43. It has been reported that NEU1 and MMP-9 form a complex with EGFR on the cell surface that is essential for the activation of EGFR 44. Here, we showed that NEU1 plays a role in mutant p53-mediated NSCLC cell migration. It is conceivable that NEU1 could be a potential therapeutic target for NSCLC patients harboring mutant p53 and EGFR.
